# Changes of Sublingual Microcirculation during the Treatment of Severe Diabetic Ketoacidosis

**DOI:** 10.3390/jcm13061655

**Published:** 2024-03-14

**Authors:** Vlasta Krausova, David Neumann, Jaroslav Skvor, Pavel Dostal

**Affiliations:** 1 Department of Pediatrics, Masaryk Hospital, Krajska Zdravotni, 40113 Usti nad Labem, Czech Republic; 2 Faculty of Medicine in Hradec Kralove, Charles University, 50003 Hradec Kralove, Czech Republic; 3 Department of Pediatrics, Trutnov Regional Hospital, 54101 Trutnov, Czech Republic; 4 Department of Pediatrics, Faculty of Medicine in Hradec Kralove, Charles University, 50003 Hradec Kralove, Czech Republic; 5 Department of Pediatrics, University Hospital Hradec Kralove, 50005 Hradec Kralove, Czech Republic; 6 Department of Anaesthesiology and Intensive Care Medicine, Faculty of Medicine in Hradec Kralove, Charles University, 50003 Hradec Kralove, Czech Republic; 7 Department of Anaesthesiology and Intensive Care Medicine, University Hospital Hradec Kralove, 50005 Hradec Kralove, Czech Republic

**Keywords:** sublingual microcirculation, diabetic ketoacidosis, hypertension

## Abstract

**Introduction:** Diabetic ketoacidosis (DKA) is associated with volume depletion and hemodynamic alterations. Changes in systemic microcirculation during DKA have not been described so far. **Methods:** In this case report, we describe the evolution of sublingual microcirculatory changes, monitored using sidestream dark field (SDF) imaging during the treatment of severe diabetic ketoacidosis in a 13-year-old girl. The patient presented a pH of 6.84, a glycemia level of 27.2 mmol/L, a ketonemia level of 5.6 mmol/L, a base excess of −29.4 mmol/L, hypernatremia, hyperosmolality due to acute gastritis, and a malfunction of the glucose sensor. Sublingual microcirculation measurements using an SDF probe were initiated 60 min after the initiation of treatment, which was then repeated 2, 3, 4, 6, 12, and 24 h after treatment initiation, as well as on the day of discharge. **Results:** Substantial alterations of microvascular perfusion parameters, both total and small vessel densities, perfused vessel densities, and the DeBacker score, were observed during the first 6 to 12 h of treatment. The degree of microcirculatory alteration was strongly negatively correlated with calculated osmolality, sodium levels, ketone and lactate levels, and blood pressure values. **Conclusions:** DKA is, in its complexity, associated with a serious microcirculatory alteration. SDF imaging provides insight into the severity of the patient’s microcirculatory alteration and its evolution during treatment.

## 1. Introduction

Diabetic ketoacidosis (DKA) is a grievous acute complication of type 1 diabetes mellitus (T1D) that occurs when there is a lack of insulin in the body [1,2]. DKA is associated with volume depletion as a result of osmotic diuresis, vomiting, and hyperventilation [2], although its gradation does not correlate well with other patients’ clinical characteristics or laboratory findings [3]. In clinical studies, the mean dehydration was 5.2% and 5.7% of the bodyweight, respectively [4,5]. More severe dehydration was associated with a new onset of diabetes, higher blood urea nitrogen, lower pH, higher anion gap, and diastolic hypertension [5]. Clinical assessment was shown to be a poor predictor of the severity of dehydration and overestimated the dehydration in 67% of patients; therefore, it is recommended to assume moderate dehydration and to make adjustments in fluid therapy according to the clinical response [6]. 

During DKA, systemic hypotension due to volume depletion and vasodilation associated with ketoacidosis should theoretically occur [7]. On the contrary, some patients present hypertension [3]. Unfortunately, a loss of coherence between macrohemadynamic parameters and microcirculation may occur in hemodynamically unstable patients [8], and several mechanisms leading to impaired microcirculation due to a neurohumoral response to hyperosmolarity [9] could also be involved during DKA.

Microcirculation abnormalities have been known to correlate with organ dysfunction and mortality in various pathological situations, both in adults and in children, such as in sepsis and other hemodynamically compromised patients [8,10,11,12,13,14,15]. Changes in the microcirculation can be detected in the initial stages of the disease, and can persist even after normalization of macrohemodynamic parameters [10,16,17,18]. Thus, microcirculation monitoring is advised in patients diagnosed with circulatory shock [16,18]. 

Microcirculatory changes can be observed via direct visualization of the sublingual microcirculation by handheld vital microscopes, such as devices that utilize sidestream dark field imaging (SDF). These devices illuminate the tissue in depth (up to 3 mm, according to the manufacturer) by emitting green light, which is absorbed by the hemoglobin of the red blood cells in the surface vessels. This leads to the visualization of arterioles, capillaries, and venules containing erythrocytes [17,18]. SDF imaging has been used to study changes in microcirculation under various clinical conditions in animal and human studies, and is also a well-validated modality used to observe microcirculation [11,18,19]. This method was also recently tested for the early noninvasive detection of diabetic nephropathy [20]. 

In this case report, we describe the evolution of microcirculatory changes monitored using sidestream dark field imaging during the treatment of a child subject with severe diabetic ketoacidosis.

## 2. Case Presentation

A 13-year old girl with severe diabetic ketoacidosis was diagnosed with T1D a year and a half earlier. She was treated with multiple daily doses of subcutaneous insulin. She had unsatisfying home treatment results with HbA1c 63 mmol/mol measured from blood samples taken on admission. The patient presented symptoms of acute gastritis and severe ketoacidosis. She was somnolent and hyperventilating, with a pH of 6.84, glycemia levels of 27.2 mmol/L, ketonemia levels of 5.6 mmol/L, and a base excess of −29.4 mmol/L, corrected sodium levels of 155 mmol/L, and a calculated hyperosmolality of 324 mOsmol/kg. The continuous glucose sensor was malfunctioning. Fluid therapy and therapy with continuous insulin, according to current recommendations [21], were initiated. After an explanation of the procedure and parents’ informed consent, sublingual microcirculation measurements were initiated. The recorded data were not used to adjust the treatment. The continuous intravenous insulin application lasted two days. On the third day, the patient was transferred to a standard ward with adjusted multiple subcutaneous insulin doses, and the patient was discharged on the fourth day.

**Figure 1:**
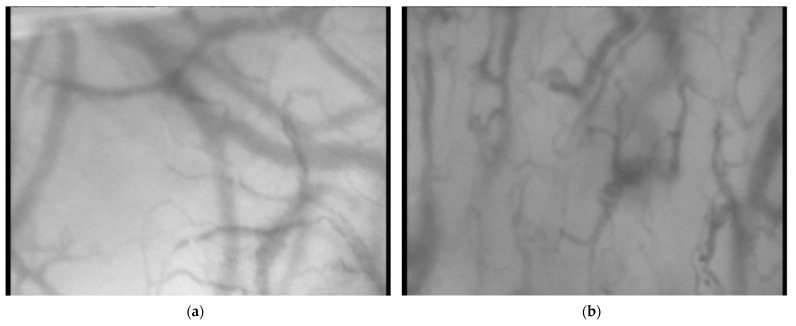
Sample pictures taken from recorded videoclips during DKA (**a**) and after DKA resolution (**b**).

Sublingual microcirculation measurements using an SDF probe (MicroScan; MicroVision Medical, Amsterdam, The Netherlands) were performed in a supine position and initiated 60 min after the initiation of treatment. A total of three video clips of 20 s each from different parts of the sublingual region were recorded immediately after each other. The measurements were repeated 2, 3, 4, 6, 12, and 24 h after the treatment initiation, as well as on the day of discharge from the hospital by a single examiner. At the time of blood sampling, the acid–base balance, glycemia levels, and ketonemia levels from capillary arterialized blood were examined. Sample pictures of sublingual microcirculation from recorded video clips during and after the resolution of DKA are presented in Figure 1.

The recorded videos were processed offline using AVA V3.0 software (AMC, University of Amsterdam, Amsterdam, The Netherlands) by one trained and experienced evaluator. The three best and most stable parts of each video clip were analyzed. A 20 μm cut-off was used to separate small vessels (mostly capillaries) from large vessels (mostly venules). The following microcirculatory parameters were analyzed: The total small-vessel density (SVD) and all-vessel density (TVD) were defined as the total length of the respective vessels inside the image divided by the total area of the image. Small vessels were defined as those with diameters ≤ 20 μm [17,18]. The DeBacker score, given in mm^−1^, was defined as the number of vessels crossing three arbitrary horizontal and three vertical equidistant lines (drawn on the screen) divided by the total length of the lines [17]. The perfused small vessel density (PSVD) and the perfused vessel density (PVD) were obtained via the multiplication of the SVD and TVD by the respective proportion of perfused vessels [17]. The normal ranges of these parameters in children were recently published [22]. 

The recorded parameters included gender, age, height, weight, non-invasively measured blood pressure, heart rate, venous blood osmolality, urea, creatinine, C-reactive protein (CRP), acid–base balance, pH, PaO_2_, PaCO_2_, base excess, actual bicarbonate, sodium, potassium, chlorides, and lactate. A point-of-care glucometer which allowed for ketonemia measurements was used for sampling. The corrected sodium level was calculated using a calculator available at https://www.mdcalc.com/calc/50/sodium-correction-hyperglycemia, accessed on 12 December 2023, according to Hillier et al. [23]. 

The anthropometric and baseline laboratory parameters of the patient are summarized in Table 1. 

Table 2 summarizes the evolution of selected microcirculatory, macrocirculatory, and laboratory parameters during the treatment. Microcirculatory parameters reached normal values after 6 to 12 h of treatment. 

The correlations between clinical and microcirculatory parameters in this individual patient are presented in Table 3 and Table 4. They are listed according to the strength of the association between the parameter and TVD, measured using the Kendall rank correlation coefficient (the statistical software Medcalc 7.6.0/Medcalc, Ostend, Belgium/was used to perform statistical analyses).

## 3. Discussion

According to our knowledge, this is the first report describing the evolution of microcirculatory alterations in DKA using SDF imaging. 

In this patient, severe microcirculatory alteration lasting from 6 to 12 h was associated with mild hypertension and hypernatremia. The pathophysiology of hypertension during DKA has yet to be fully understood. In children, hypertension was present in up to 82% of patients during the first 6 h following admission [24]. In a recently published analysis, higher blood glucose and higher glucose-corrected sodium levels and a low baseline pH, low pCO_2_, and low Glasgow Coma Scale levels were all identified as risk factors for hypertension during DKA [3]. This patient presented stage one systolic hypertension and diastolic prehypertension [25]. Her blood pressure normalized during therapy, parallel with an improving sublingual microcirculation. Although it was suggested that hypertension might reflect a neurophysiological response to altered brainstem perfusion [26], hypertension could also be a consequence of the increased release of vasopressin, or the over-activity of the renin-angiotensin system often described in patients with DKA [27].

Principally, four types of microcirculatory alterations could be identified: type 1, heterogeneous microcirculatory flow; type 2, reduced capillary density; type 3, microcirculatory flow reduction caused by vasoconstriction or tamponade; and type 4, tissue edema [8]. In this case, reduced capillary density was the leading alteration. Several mechanisms could be involved. The endothelial and smooth muscle cells predominantly regulate the microvascular blood flow via three main mechanisms: myogenic, neurohumoral, and metabolic control. Ketoacidosis could be associated with catecholamine release [28]. The lower partial pressure of CO_2_ (pCO_2_), lactate, adenosine, and H+, and the increased partial pressure of oxygen (pO_2_) are associated with vasoconstriction. Precapillary sphincters also constrict in response to concentrations of potassium, magnesium, osmolarity, and adenosine [29]. 

Hyperglycemia is well known for its lowering effect on serum sodium levels [23]. Although hyponatremia in DKA is, therefore, a more common symptom, hypernatremia can also occur. Its exact etiology is unclear, and several mechanisms related to water deficit from inadequate oral intake and free water loss have been proposed [26]. An excessive intake of carbonated carbohydrate-rich beverages has also worsened hypernatremia [30,31]. In our patient, sodium levels, especially glucose-corrected sodium, were increased, probably due to vomiting, a low oral intake of fluids, and urinary water loss. The degree of hypernatremia was strongly associated with the severity of microcirculatory alteration. Although we cannot generalize the relationships observed in this single case, this observation adds a further mechanistic explanation for the previously described evolution of peripheral gangrene in children with hypernatremic dehydration [32]. High sodium levels were shown to augment vasoconstrictor responses to catecholamines [33]. Hyperosmolarity also increases the release of vasopressin with the potential to alter microcirculation [9]. Hyperosmolarity, both due to high glucose levels and hypernatremia, could also induce vasoconstriction through the Rho/Rho-kinase signaling pathway [34]. The development of hypertension and microcirculatory alteration share several pathophysiological mechanisms, and both could reflect the intensity of the neurohumoral response to water depletion and hyperosmolarity during DKA. 

The monitoring of microcirculatory alterations during DKA using SDF imaging is of potential clinical interest. This method of microcirculatory monitoring is noninvasive, repeatable, and validated under several clinical conditions. It may improve our understanding of DKA pathophysiology and our ability to predict the development of DKA complications early on [20]. Identifying microcirculatory alterations and the responses to treatment could enable personalized fluid therapy for groups of patients [35]. In theory, assessing the severity of microcirculatory alteration during DKA could also help distinguish non-genetic influences of the development of long-term complications of diabetes, such as retinopathy, neuropathy, or nephropathy. More studies on patients with both mild and severe forms of DKA, both with/without hypernatremia and hypertension, are needed to improve our understanding of DKA as it pertains to microcirculatory alteration and the clinical utility of SDF imaging.

## 4. Conclusions

In a child with mild hypertension, DKA with hypernatremia was associated with a serious alteration of sublingual microcirculation. SDF ismaging provided insight into the severity of the patient’s microcirculatory alteration and its evolution during treatment.

## Figures and Tables

**Table 1:** Anthropometric and baseline laboratory parameters.

Parameter	Value
Age	13
Height (cm)	153
Weight (kg)	45.5
Heart rate (/min)	130
SAP (mmHg)	128
DAP (mmHg)	77
MAP (mmHg)	94
SpO_2_ (%)	100
pH	6.841
pCO_2_ (kPa)	3.23
pO_2_ (kPa)	15
HCO_3_^−^ (mmol/L)	5.8
Base excess (mmol/L)	−29.7
Glucose (mmol/L)	27.15
Ketones (mmol/L)	5.6
HbA1c (mmol/moL)	63
CRP (mg/L)	<1
Calculated Osmolality (mOsmol/kg)	324
Urea (mmol/L)	4.6
Creatinine (mmol/L)	108
Sodium (mmol/L)	146
Chlorides (mmol/L)	113
Potassium (mmol/L)	4.3
Lactate (mmol/L)	4.1

SAP, Systolic arterial pressure; DAP, Diastolic arterial pressure; MAP, Mean arterial pressure; SpO_2_, O_2_ saturation of hemoglobin; HbA1c, glycated hemoglobin; CRP, C-reactive protein.

**Table 2:** Evolution of selected microcirculatory and laboratory parameters.

Parameter	Time (h)							
	1	2	3	4	6	12	24	51
TVD (mm/mm^2^)	6.11	7.04	9.58	8.92	10.87	10.12	11.11	10.30
SVD (mm/mm^2^)	1.37	2.88	5.11	5.23	8.35	8.64	8.50	7.82
PVD (mm/mm^2^)	5.97	6.80	9.53	8.79	10.58	10.06	11.09	10.18
PSVD (mm/mm^2^)	1.32	2.73	5.06	5.23	8.06	8.59	8.47	7.68
DeBS (1/mm)	4.04	4.70	6.13	5.81	6.37	6.38	6.99	6.29
SAP (mmHg)	128	127	124	118	114	117	116	108
DAP (mmHg)	77	71	60	65	58	56	64	64
MAP (mmHg)	94	90	81	83	77	76	81	79
pH	6.84	7.04	7.09	7.16	7.19	7.28	7.31	7.47
pO_2_ (kPa)	15.0	15.1	15.2	15.2	12.9	7.7	9.2	9.0
Glucose (mmol/L)	27.15	14.9	13.8	12.4	13.5	14	15.2	10.8
Ketones (mmol/L)	5.6	4.5	4.4	3.9	2.6	2.3	2.6	0
GCNa (mmol/L)	155	149	150	146	145	147	144	144
Lactate (mmol/L)	4.1	1.3	1.3	1.0	0.8	0.9	0.7	2.2
Calculated Osmolality (mOsm/kg)	324	310	310	303	302	304	299	303

TVD, total vessel density; SVD, small vessel density; PVD, perfused vessel density; PSVD, perfused small vessel density; DeBS, DeBacker’s score; SAP, systolic arterial pressure; DAP, diastolic arterial pressure; MAP, mean arterial pressure; pO_2_, parcial O_2_ pressure; GCNa, glycemia corrected sodium.

**Table 3:** Relationship between laboratory and microcirculatory parameters.

	TVD (mm/mm^2^)	SVD (mm/mm^2^)	PVD (mm/mm^2^)	PSVD (mm/mm^2^)	DeBS (1/mm)
Calcutated osmolality (mOsm/kg)	−0.71 *p* = 0.009	−0.57 *p* = 0.035	−0.71 *p* = 0.009	−0.57 *p* = 0.035	−0.57 *p* = 0.035
GCNa (mmol/L)	−0.69	−0.55	−0.69	−0.55	−0.55
	*p* = 0.012	*p* = 0.044	*p* = 0.012	*p* = 0.044	*p* = 0.044
Sodium (mmol/L)	−0.67	−0.67	−0.67	−0.67	−0.67
	*p* = 0.015	*p* = 0.015	*p* = 0.015	*p* = 0.015	*p* = 0.015
Ketones (mmol/L)	−0.62	−0.76	−0.62	−0.76	−0.62
	*p* = 0.023	*p* = 0.006	*p* = 0.023	*p* = 0.006	*p* = 0.023
Lactate (mmol/L)	−0.62	−0.62	−0.62	−0.62	−0.62
	*p* = 0.023	*p* = 0.023	*p* = 0.023	*p* = 0.023	*p* = 0.023
pO_2_ (kPa)	−0.26	−0.47	−0.26	−0.47	−0.40
	*p* = 0.314	*p* = 0.078	*p* = 0.314	*p* = 0.078	*p* = 0.131
Glucose (mmol/L)	−0.21	−0.21	−0.21	−0.21	−0.07
	*p* = 0.387	*p* = 0.387	*p* = 0.387	*p* = 0.387	*p* = 0.711
Chlorides (mmol/L)	0.00	0.14	0.00	0.14	0.00
	*p* = 0.846	*p* = 0.846	*p* = 0.846	*p* = 0.846	*p* = 0.846
Glucose dose (mg/kg/min)	0.16	0.24	0.16	0.24	0.16
	*p* = 0.675	*p* = 0.485	*p* = 0.675	*p* = 0.485	*p* = 0.675
pCO_2_ (kPa)	0.33	0.20	0.33	0.20	0.33
	*p* = 0.452	*p* = 0.707	*p* = 0.452	*p* = 0.707	*p* = 0.452
HCO_3_^−^ (mmol/L)	0.60	0.47	0.60	0.47	0.60
	*p* = 0.133	*p* = 0.260	*p* = 0.133	*p* = 0.260	*p* = 0.133
BE (mmol/L)	0.60	0.47	0.60	0.47	0.60
	*p* = 0.133	*p* = 0.260	*p* = 0.133	*p* = 0.260	*p* = 0.133
pH	0.71	0.71	0.71	0.71	0.71
	*p* = 0.217	*p* = 0.197	*p* = 0.205	*p* = 0.195	*p* = 0.225

Results are presented as a value of the Kendall’s Tau and its *p*-value. TVD, total vessel density; SVD, small vessel density; PVD, perfused vessel density; PSVD, perfused small vessel density; DeBS, DeBacker’s score; pCO_2_, partial CO_2_ pressure; pO_2_, partial O_2_ pressure; HR, heart rate; BE, base excess; GCNa, glycemia corrected sodium.

**Table 4:** Relationship between the macrocirculatory and microcirculatory parameters.

	TVD (mm/mm^2^)	SVD (mm/mm^2^)	PVD (mm/mm^2^)	PSVD (mm/mm^2^)	DeBS (1/mm)
SAP (mmHg)	−0.71	−0.57	−0.71	−0.57	−0.57
	*p* = 0.009	*p* = 0.035	*p* = 0.009	*p* = 0.035	*p* = 0.035
MAP (mmHg)	−0.62	−0.76	−0.62	−0.76	−0.76
	*p* = 0.023	*p* = 0.006	*p* = 0.023	*p* = 0.006	*p* = 0.006
DAP (mmHg)	−0.55	−0.69	−0.55	−0.69	−0.69
	*p* = 0.044	*p* = 0.012	*p* = 0.044	*p* = 0.012	*p* = 0.012
HR (/min)	−0.55	−0.40	−0.55	−0.40	−0.55
	*p* = 0.044	*p* = 0.131	*p* = 0.044	*p* = 0.131	*p* = 0.044

Results are presented as a value of the Kendall’s Tau and its *p*-value. TVD, total vessel density; SVD, small vessel density; PVD, perfused vessel density; PSVD, perfused small vessel density; DeBS, DeBacker’s score; SAP, systolic arterial pressure; DAP, diastolic arterial pressure; MAP, mean arterial pressure.

## Data Availability

The original contributions presented in the study are included in the article. Further inquiries can be directed to the corresponding author.
